# Sequence Analysis of Lysozyme C from the Scorpion Mesobuthus Eupeus Venom Glands Using Semi-Nested RT-PCR

**Published:** 2011-10-01

**Authors:** M Baradaran, A Jolodar, A Jalali, Sh Navidpour, F Kafilzadeh

**Affiliations:** 1 Toxicology Research Center, Jundishapur University of Medical Sciences, Ahvaz, Iran; 2 Department of Basic Sciences, Faculty of Veterinary Medicine, Shahid Chamran University of Ahvaz, Ahvaz, Iran; 3 Department of Pharmacology and Toxicology, School of Pharmacy, Toxicology Research Center, Jundishapur University of Medical Sciences, Ahvaz, Iran; 4 Veterinary Parasitology Department of Razi Vaccine and Serum Research Institute, Karaj, Iran; 5 Azad Islamic University, Jahrom Branch, Jahrom, Iran

**Keywords:** C-type lysozyme, Scorpion, Mesobuthus eupeus, Antimicrobial protein

## Abstract

**Background:**

Lysozyme is an antimicrobial protein widely distributed among eukaryotes and prokaryotes and take part in protecting microbial infection. Here, we amplified cDNA of MesoLys-C, a c-type lysozyme from the most common scorpion in Khuzestan Province, Southern Iran.

**Methods:**

Scorpions of Mesobuthus eupeus were collected from the Khuzestan Province. Using RNXTM solution, the total RNA was extracted from the twenty separated venom glands. cDNA was synthesized with extracted total RNA as template and modified oligo(dT) as primer. In order to amplify cDNA encoding a lysozyme C, semi-nested RT-PCR was done with the specific primers. Follow amplification, the fragment was sequenced.

**Results:**

Sequence determination of amplified fragment revealed that MesoLys-C cDNA had 438 bp, encoding for 144 aa residues peptide with molecular weight of 16.702 kDa and theoretical pI of 7.54. A putative 22-aminoacids signal peptide was identified. MesoLys-C protein was composed of one domain belonged to c-type lysosyme/ alphalactalbumin.

**Conclusion:**

Multiple alignment of MesoLys-C protein with the related cDNA sequences from various organisms by ClustalW program revealed that some of the conserved residues of other c-type lysosymes were also seen in MesoLys-C. However, the comparison suggested that Mesobuthus eupeus of Khuzestan and east Mediterranean Mesobuthus eupeus belonged to different subspecies.

## Introduction

Lysozyme is a widespread antimicrobial protein occurring in insects, vertebrates, plants and microorganisms. Lysozymes are muramidases that hydrolyse the β-1,4 glycosidic linkage in the N-acetyl glucosamine and N-acetyl muramic acid residues in the peptidoglycan layer of the bacterial cell walls and cause their lysis.[1] Lysozymes are classified into three major types including chicken type (c-type), goose type (gtype) and invertebrate type (i-type). The c-type lysozyme was found in many organisms including viruses, bacteria, plants, reptiles, birds and mammals.[2][3] Ctype lysozymes were also reported in different classes of the arthropoda phylum, namely in several species of lepidopteran, dipteran, isopteran and hemipteran insects, in arachnids and the crustaceans.[4]

A subset of arthropod venoms are complex mixtures of highly evolved peptidic libraries with toxin activities that include antimicrobial pore forming and ion channel.[5] These peptides have the potential to combat cancer tumors and a variety of bacterial and fungal infections.[6] So far, several c-type lysozymes were identified and characterized from different organisms like scorpion Scorpiops jendeki,[7] silkworms of Bombyx mori[8] and Antheraea mylitta,[9] Asian corn borer of Ostrinia furnacalis,[10] and banana prawn of Fenneropenaeus merguiensis.[3] Mesobuthus eupeus is one of the most frequent scorpion from Mesobuthus species and belongs to Buthidae family. This scorpion is reported from the most area of Iran, specially Khuzestan Province.[11] In the present study, cDNA of MesoLys-C was amplified and characterized from scorpion Mesobuthus eupeus from Khuzestan Province.

## Materials and Methods

Scorpions Mesobuthus eupeus were collected from the Khuzestan Province of Iran. Twenty separated venom glands were used for total RNA extraction.

Total RNA was extracted from the venom glands of scorpions using RNA™ (Cinnagen, Iran) according to the manufacture procedure. RNA pellet was dissolved in DEPC-ddH2O and used for cDNA synthesis immediately. cDNA was synthesized with the extracted total RNA as template and ModT (modified oligodT) (5´-gggtctagagctcgagtcacttttttttttttttttt-3´) as primer. ModT was added to extracted.

RNA and incubated in 70ºC for 5 minutes and then immediately on ice for 2 minutes. Then, 5X buffer, dNTP, Ribolock, reverce transcriptase enzyme and ddH2O were added to samples and incubated for 60 minutes in 42ºC. Samples were incubated 10 minutes in 70ºC and immediately on ice.

Amplification of Lys-c cDNA was performed using semi-nested RT-PCR strategy. specific primers used in semi-nested RT-PCR were designed according to cDNA sequence of lysozyme C from Mesobuthus gibbusus.[12] The first round of PCR was performed using ModT-R (5 ´-cccagatctcgagctcagtg-3´), lyc-F (5 ´- gcgcggatccaagatggctttcaagttttcatt-3 ´) primers and synthesized cDNA as template. Second round of PCR was performed using lyc-F and lyc-R (5´- gcgcaagctttacagttgttatcattgataaattg-3´) primers. PCR products of initial amplification were used as template for the secound round of amplification. The PCR conditions for both rounds were 35 cycles with denaturation at 94ºC (40 sec), anealing at 56ºC (90 sec) and extention at 72ºC (1 min) with a initial denaturation at 95ºC (5 min) and final extention at 72ºC (10 min). Amplification products were separated by agarose gel electrophoresis and visualized by UV transilluminatore.

The amplified cDNA fragments were purified from agarose gel by QIAquick agarose gel extraction kit (Qiagen, Germany) and then sent to Kowsar Biotech Company for nucleotide sequencing.

Sequence was compared with GeneBank database using the BLAST software from NCBI site (http://www.ncbi.nlm.nih.gov). The tool software available at the ExPaSy website (http://ca.expasy.org/ tools/pi_tool.html) was used to convert nucleotide sequence to amino acid. The molecular weight and theoretical pI was estimated using ProtParam tool (http://www.expasy.org/tools/protparam.html). The signal peptide was predicted by SignalP (http://www.cbs.dtu.dk/services/SignalP/). Multiple sequence alignments were done using the CLUSTAL_W program and edited with the BOXSHADE software (http://www.ch.embnet.org/software/BOX_form.html). The SBASE online software (http://hydra.icgeb.trieste.it/sbase/) was used to determine the conserved domains.

## Results

**Fig. 1:**
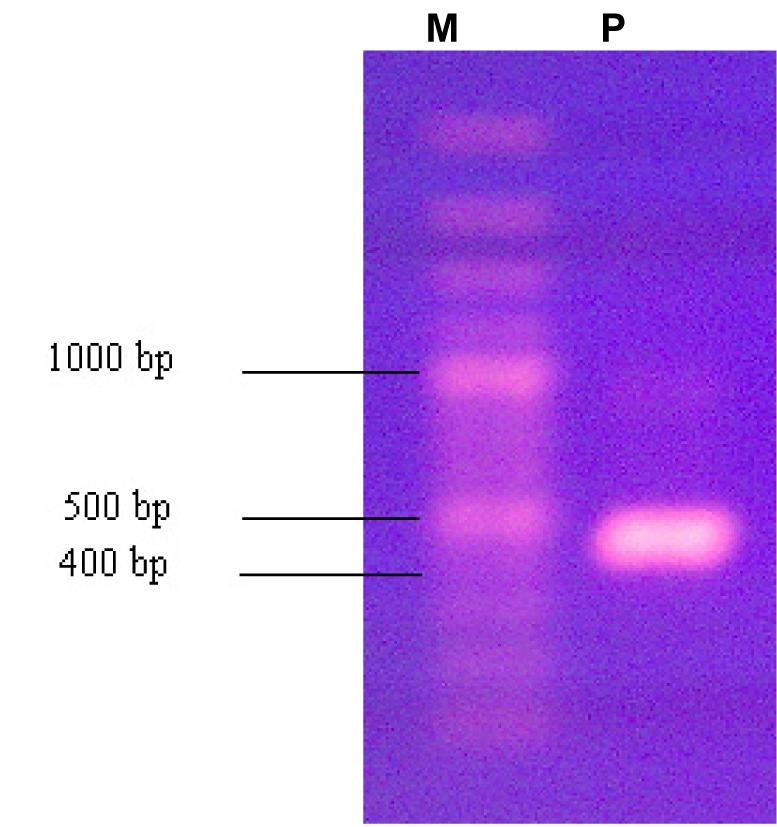
PCR amplification of theLys-C cDNA from Mesubuthus eupeus. Lane M: DNA marker, Lane P: production of amplification of the Lys-C cDNA from Mesubuthus eupes

Figure 1 shows PCR amplification of theLys-C cDNA from Mesobuthus eupeus. As shown in this figure, Semi-nested RT-PCR amplified a fragment in range about 450 bp.

**Fig. 2:**
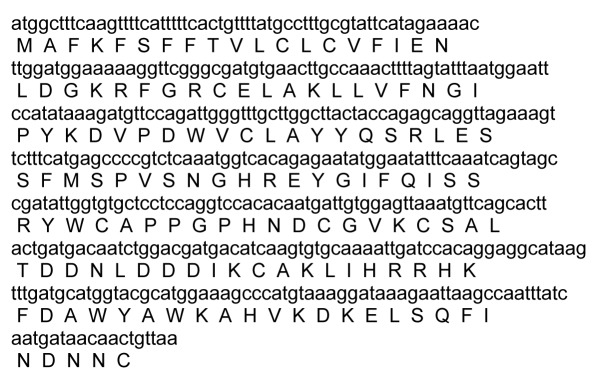
cDNA and amino acid sequences of the MesoLys-C

**Fig. 3:**
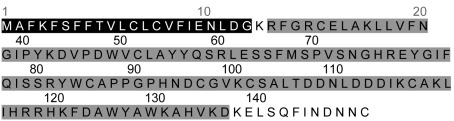
Signal peptide and glycoside hydrolase domain of MesoLys-C. The residues corresponding to the signal peptide are indicated in black and the residues corresponding to the glycoside hydrolase domain is indicated in gray.

According to the sequencing results, the peptide coding sequence was 438 bp in length, encoding for 144 aa residues peptide (Figure 2) with molecular weight 16.702 kDa and theoretical pI of 7.54. Comparative details of theoretical pI and molecular weight of identified c-type lysozymes of animials were shown in Table 1. A putative 22-amino-acids signal peptide (Figure 3) was identified and the lysine at position 23 was assumed to represent the start of the mature protein.

**Table 1:** Molecular weight and theoretical pI of c-type lysozyme from Khuzestanian Mesobuthus eupeus and ctype lysozyme from 15 other organisms that their amino acid sequences are indicated in  Figure 4

**Organism**	**Molecular Weight (kDa)**	**Theoretical pI**
*Mesobuthus eupeus* (Khuzestan Province, Iran)	160702	7.54
*Mesobuthus gibbosus*	12.2	8.26
*Mesobuthus cyprius*	12.216	8.26
East Mediterranean *Mesobuthus eupeus*	12.253	7.76
*Dermacentor variabilis*	15.732	9.97
*Ixodes scapularis*	15.441	9.97
*Anopheles gambiae*	16.538	8.92
*Simulium nigrimanum*	15.877	8.95
*Spodoptera exigua*	16.235	8.92
*Manduca sexta*	16.087	8.91
*Bombyx mori*	15.007	8.98
*Ornithodoros moubata*	16.227	8.07
*Drosophila melanogaster*	15.65	5.47
*Mus musculus*	16.794	9.55
*Chicken Gallus gallus*	16.239	9.36

**Fig. 4:**
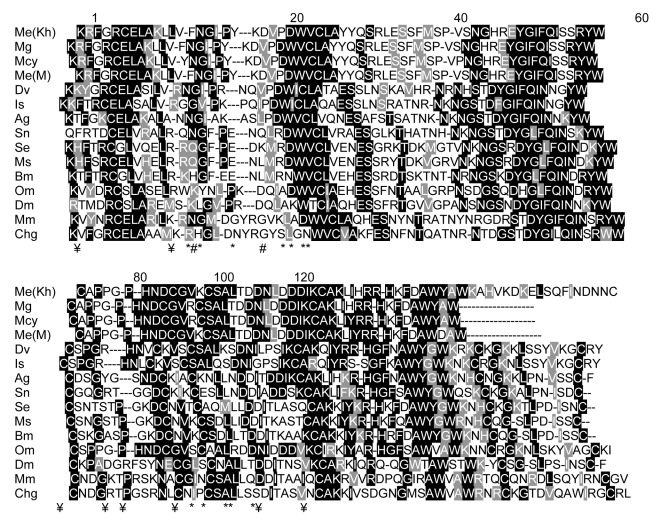
Sequence alignment of MesoLys-C with c-type lysozyme sequence from the chicken and mouse from vertebrates, three species of scorpions and some other arthropoda. Gaps are indicated by (-), conserved cysteines are indicated by (¥), the position of two catalytic residues is indicated by (#), the position of other active site residues is indicated by (*). Homologous amino acids are shaded in grey and fully conserved amino acids are shaded in black. Me (Kh): Mesobuthus eupeus from Khuzestan, Mg: Mesobuthus gibbosus(CAE55016), Mcy: Mesobuthus cyprius (CAE55013), Me(M): east Mediterranean Mesobuthus eupeus (CAE55006), Dv: Dermacentor variabilis (AAO23571), Is: Ixodes scapularis (XP_002399439), Ag: Anopheles gambiae (AAC47326), Sn: Simulium nigrimanum (ACZ28238), Se: Spodoptera exigua (AAP03061), Ms: Manduca sexta (AAB31190), Bm: Bombyx mori (NP_001037448), Om: Ornithodoros moubata (AAL17868), Dm: Drosophila melanogaster (ABK57077), Mm: Mus musculus (NP_990612), Chg: Chicken Gallus gallus (NP_038618).

Comparison of the cDNA fragment with the GeneBank database revealed that the amino acid sequence of MesoLys-c was highly homologous with ctype lysozymes of other scorpions and arthropods. It is suggested that MesoLys-c belongs to the c-type lysozyme family. Conserved domains of MesoLys-c were predicted using SBASE online software. As shown in Figure 3, MesoLys-c had one conserved domain. This domain was glycoside hydrolase belonging to c-type lysozyme/alphalactalbumin.

In Figure 4, the amino acid sequence of MesoLysc was aligned with c-type lysozyme from three species of scorpions including Mesobuthus gibbosus, Mesobuthus cyprius, east Mediterranean Mesobuthus eupeus,[12] nine other arthropoda including, Dermacentor variabilis,[13] Ixodes scapularis, Anopheles gambiae, [14] Simulium nigrimanum,[15] Spodoptera exigua,[16] Manduca sexta,[17] Bombyxmori,[18] Ornithodoros moubata, [19] Drosophila melanogaster, and two organisms of vertebrates including chicken Gallus gallus,[20] and mouse Mus musculus.[21] As shown in Figure 4, MesoLys-c and c-type lysozyme of other scorpions had 6 conserved cysteines residues (Cys6 , Cys27 , Cys61 , Cys70 , Cys74 , Cys88 ), while c-type lysozyme of other organisms had 8 conserved cysteine residues (Cys6 , Cys27 , Cys61 , Cys70 , Cys74 , Cys88 , Cys108, Cys120). There was a triple-peptide (YRG) in lysozyme of Mus musculus and chicken that was absent in other organisms.

**Fig. 5:**
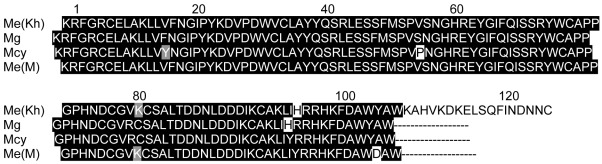
Sequence alignment of MesoLys-C with 3 other scorpion species lysozymes. Gaps are indicated by (-). Homologous amino acids are shaded in grey and fully conserved amino acids are shaded in black. Me (Kh): Mesobuthus eupeus from Khuzestan, Mg: Mesobuthus gibbosus, Mcy: Mesobuthus cyprius, Me (M): east Mediterranean Mesobuthus eupeus

The amino acid sequence of MesoLys-c was aligned with c-type lysozyme from scorpions Mesobuthus gibbusus, Mesobuthus cyprius and east Mediterranean Mesobuthus eupeus.[12] As shown in Figure 5, amino acid sequences of c-type lysozymes in different scorpions were very similar and there were small differences between sequences from different scorpions. According to Figure 5, MesoLys-c had unsimilarity with c-type lysozyme of Mesobuthus gibbosus in one amino acid (Arg77 in M. gibbosus instead of Lys77 in MesoLys-c) and with c-type lysozyme of M. cyprius in 4 amino acids and of east Mediterranean M. eupeus in 2 amino acids. The residues Phe14, Ser44, Lys73 and His93 of MesoLys-c were Tyr , phe , Arg and Tyr in c-type lysozyme of M. cyprius, respectively. Unsimilarities in amino acid sequence between MesoLys-c and ctype lysozyme of east Mediterranean M. eupeus occured at His93 and Tyr102 in MesoLys-c which were Tyr and Asp, in c-type lysozyme of east Mediterranean M. eupeus, respectively.

## Discussion

C-type lysozyme from the venom of scorpions such as Tityus stigmurus, east Mediterranean Mesobuthus eupeus, Mesobuthus gibbusus, Mesobuthus cyprius and Scorpiops jendeki were previously sequenced.[12][22] However c-type lysozyme of scorpions venom was not studied in Iranian scorpions fauna. In this study, MesoLys-C was identified from venom gland of Mesobuthus eupeus of Khozestan Province. The amino acid sequence of MesoLys-C was compared in Figure 3 to c-type lysozymes from several organisms. Previous studies have revealed that c-type lysozymes had 8 conserved cysteines residues.[9][10] As shown in Figure 3, MesoLys-C and c-type lysozyme of other scorpions had 6 conserved cysteines residues whilst c-type lysozyme of other organisms had 8 conserved cysteines residues. It suggests that c-type lysozyme of scorpion form 3 disulfide bridge whilst c-type lysozyme of the other organisms form 4 disulfide bridge in final conformation.

Examination on chicken lysozyme revealed that the active site of this protein defined by 14 residues.[4] Through comparison with chicken lysozyme, the active site of MesoLys-C could be defined by residues Tyr31, Gln32, Ser33, Pro42, Glu49, Gln54, Ser56, Tyr59, Trp60, Ile92, Arg95, Ala100, Trp101and His107. In chicken lysozyme, the residues Glu32 and Asp49 were implicated to have direct role in catalysis.[9][10]The comparison that was done in this study revealed that the residues of catalytic site in c-type lysozyme of scorpions were different from those of chicken and other organisms. Moreover, in all of them one of catalytic site residues was glutamic acid. It is suggested that glutamic acid has more important role rather than second residue in action of this enzyme. This comparison revealed lack of triple-peptide (YKG) in all c-type lysozyme except ctype lysozyme of Mus musculus and chicken (of vertebrates). Since the absence of this triple-peptide did not cause inactivation of c-type lysozymes, it is obvious that this triple-peptide had no role in activity and conformation of c-type lysozymes.

Comparison of the Mesolys-C amino acid sequence with c-type lysozyme of other scorpions has revealed that the c-type lysozyme of scorpions was very similar to each other. MesoLys-C had the most similarity with the c-type lysozyme described in M. gibbosus and the least similarity with c-type lysozyme of M. cyprius. According to differences between MesoLys-C and c-type lysozyme of east Mediterranean M. eupeus, it is concluded that M. eupeus of Khuzestan Province in Southern Iran and east Mediterranean M. eupeus belong to different subspecies.
